# New Insights into the Mechanisms of Action of Topical Administration of GLP-1 in an Experimental Model of Diabetic Retinopathy

**DOI:** 10.3390/jcm8030339

**Published:** 2019-03-11

**Authors:** Joel Sampedro, Patricia Bogdanov, Hugo Ramos, Cristina Solà-Adell, Mireia Turch, Marta Valeri, Olga Simó-Servat, Carmen Lagunas, Rafael Simó, Cristina Hernández

**Affiliations:** 1 Diabetes and Metabolism Research Unit, Vall d’Hebron Research Institute, 08035 Barcelona, Spain; joel.sampedro@vhir.org (J.S.); patricia.bogdanov@vhir.org (P.B.); hugo.ramos@vhir.org (H.R.); cristina.sola@vhir.org (C.S.-A.); mireia.turch@vhir.org (M.T.); olga.simo@vhir.org (O.S.-S.); 2 Centro de Investigación Biomédica en Red de Diabetes y Enfermedades Metabólicas Asociadas (CIBERDEM), Instituto de Salud Carlos III (ICSIII), 28029 Madrid, Spain; 3 Unit of High Technology, Vall d’Hebron Research Institute, 08035 Barcelona, Spain; marta.valeri@vhir.org; 4 Ferrer Advanced Biotherapeutics, Ferrer, Diagonal 549, 08029 Barcelona, Spain; clagunas@ferrer.com; 5 Department of Medicine, Universitat Autònoma de Barcelona, 08193 Barcelona, Spain

**Keywords:** diabetic retinopathy, glucagon-like peptide-1, Retinal neurodegeneration, experimental diabetes

## Abstract

The main goals of this work were to assess whether the topical administration of glucagon-like peptide-1 (GLP-1) could revert the impairment of the neurovascular unit induced by long-term diabetes (24 weeks) in diabetic mice and to look into the underlying mechanisms. For that reason, db/db mice were treated with eye drops of GLP-1 or vehicle for 3 weeks. Moreover, db/+ mice were used as control. Studies performed in vivo included electroretinogramand the assessment of vascular leakage by using Evans Blue. NF-κB, GFAP and Ki67 proteins were analyzed by immunofluorescence (IF). Additionally, caspase 9, AMPK, IKBα, NF-κB, AKT, GSK3, β-catenin, Bcl-xl, and VEGF were analyzed by WB. Finally, VEGF, IL-1β, IL-6, TNF-α, IL-18, and NLRP3 were studied by reverse transcription-polymerase chain reaction (RT-PCR) and immunofluorescence. We found that topical administration of GLP-1 reverted reactive gliosis and albumin extravasation, and protected against apoptosis and retinal dysfunction. Regarding the involved mechanisms, GLP-1 exerted an anti-inflammatory action by decreasing NF-κB, inflammosome, and pro-inflammatory factors. In addition, it also decreased VEGF expression. Furthermore, GLP-1 promoted cell survival by increasing the anti-apoptotic protein Bcl-xl and the signaling pathway Akt/GSK3b/β-catenin. Finally, Ki67 results revealed that GLP-1 treatment could induce neurogenesis. In conclusion, the topical administration of GLP-1 reverts the impairment of the neurovascular unit by modulating essential pathways involved in the development of diabetic retinopathy (DR). These beneficial effects on the neurovascular unit could pave the way for clinical trials addressed to confirm the effectiveness of GLP-1 in early stages of DR.

## 1. Introduction

Diabetic retinopathy (DR), the most common complication of diabetes, is the primary cause of blindness among the working-age population of western countries [1].

The current treatments recommended for arresting the progression of DR (i.e., laser photocoagulation, intravitreal injections of antiangiogenic factors such as anti-vascular endothelial growth factor (VEGF) or corticosteroids, vitreoretinal surgery) are specifically addressed to very advanced stages of DR. In addition, all these treatments are quite expensive, present relevant adverse effects, and a vitreoretinal specialist is required [2]. Consequently, new treatments for treating early stages of the disease are urgently needed.

In recent years, a myriad of experimental studies have indicated that neurodegeneration is an early event in the diabetic retina and several mechanisms linking neurodegeneration and microvascular disease has been proposed [3,4,5]. In addition, the pivotal role of neurovascular unit (NVU) impairment in the pathogenesis of DR has been underlined [6]. As a result of all this evidence, the American Diabetes Association has recently defined DR as a highly specific neurovascular disease of the retina rather than a merely microvascular disease [7]. The clinical impact of this new concept will be important not only in terms of screening based on measuring retinal neurodegeneration but also in the development of drug discovery and new strategies for the early treatment of the disease.

Numerous experimental treatments based on neuroprotection have been reported in the last decade. However, only a few have been able to have an impact on microvascular disease; among them, GLP-1 receptor agonists (GLP-1RAs). In this regard, we previously reported that the topical administration of GLP-1 or GLP-1RAs using eye-drops prevented retinal neurodegeneration and early vascular leakage in db/db mice 12 weeks old [8]. In this experiment, the treatment was initiated when db/db mice were 10 weeks old, a stage in which only minimal if any diabetes-induced abnormalities in the retina could be detected [9]. Therefore, the beneficial effect of GLP-1 or GLP-1RAs was considered mainly preventative. It should be noted that topical treatment was unable to reduce blood glucose levels and, therefore, the observed effects of GLP-1 or GLP-1RAs were attributed to a local direct effect unrelated to an improvement of metabolic control.

In the present study we wanted to assess in the same model whether the topical administration of GLP-1 reverts the impairment of the NVU when retinal neurodegeneration and microvascular impairment are already well-established. In addition, the underlying mechanisms have been examined.

## 2. Experimental Section

### 2.1. Experimental Design

A total of 30 diabetic male db/db (BKS.Cg-Dock7m +/+ Leprdb/J) mice and 15 non-diabetic mice (db/+; control group) were purchased (Charles River Laboratories, Calco, Italy) for the study. Animals had free access to ad libitum food (ENVIGO Global Diet Complete Feed for Rodents, Mucedola, Milan, Italy) and filtered water. Mice were maintained under tight environmental conditions of temperature (20 °C) and humidity (60%). Moreover, they had cycles of 12 h/12 h light/darkness. In order to minimize variability, animals were randomly housed (block randomization) in groups of four mice per cage. Each cage contained absorbent bedding and nesting material (BioFresh Performance Bedding 1/8″ Pelleted Cellulose, Absorption Corp, Ferndale, WA, USA).

### 2.2. Interventional Study

When the mice were aged 21 weeks, GLP-1 eye-drops (*n* = 15) or vehicle eye-drops (*n* = 15) were randomly administered directly onto the superior corneal surface of each eye using a syringe. One drop (5 µL) of GLP-1 (2 mg/mL), or vehicle (5 µL phosphate-buffered saline (PBS), pH 7.4) was administered twice daily for three weeks in each eye. On the last day (24 weeks of age), one drop of either GLP-1 or vehicle was administered to the eyes 1 h before euthanasia. The evaluation of the results was performed by investigators unaware of treatment received by the mice.

This study was approved by the Animal Care and Use Committee of VHIR (Vall d’Hebron Research Institute). All the experiments were performed in accordance with the tenets of the European Community (86/609/CEE) and the Association for Research in Vision and Ophthalmology (ARVO).

### 2.3. Electroretinogram

Full-field electroretinogram recordings were measured using the Ganzfeld ERG platform (Phoenix Research Laboratories, Pleasanton, CA, USA). Animals were dark adapted for at least 8 h prior to ERG recording and then anesthetized with isoflurane. Tropicamide (1%) was applied to each eye prior to the test. A cutaneous ground electrode was placed near the base of the tail, a needle electrode was placed cutaneously on the head between the two eyes and a cornel electrode was placed near each eye. Carboxymethylcellulose (1%) drops were applied to the interior surface of the contact lens electrodes prior to their placement on the eyes. The ERG parameters were measured as defined by the International Society for Clinical Electrophysiology of Vision [10].

### 2.4. Tissue Processing

The mice were killed by cervical dislocation. For mRNA and protein assessments the retinas were separated immediately after enucleation, frozen in liquid nitrogen, and stored at −80 °C. Retinas used for immunohistochemical analysis were obtained from mice after transcardial perfusion with p-formaldehyde 4%. In these cases, intraperitoneal injection of anaesthesia (1 mL ketamine and 0.3 mL xylazine) was previously administered.

### 2.5. RNA Isolation and Quantitative Reverse Transcription Polymerase Chain Reaction (RT-PCR) Assay

Total RNA from mice was extracted using Trizol^®^ reagent (Invitrogen, Madrid, Spain) according to the manufacturer’s protocol. Then, RNA samples were treated with DNase (Qiagen, Madrid, Spain) to remove genomic contamination and purified on a RNeasy MinElute column (Qiagen, Madrid, Spain). RNA quantity was measured on a Nanodrop spectrophotometer, and integrity was determined on an Agilent 2100 Bioanalyzer. The single strand cDNA was synthesized as described in Prime Script^®^ RT Master Mix kits. Real-time reverse transcription polymerase chain reaction (RT-PCR) was performed using SYBR Green PCR Master Mix (Applied Biosystems, Warrington, UK) using the 7.900 HT Sequence Detection System in 384-well optical plates with specific primers displayed in Table 1.

For each sample, RT-PCRs were performed in triplicate and relative quantities were calculated using ABI SDS 2.0 RQ software, analyzed by the 2−ΔΔCt method, and given as ratio compared to β-actin as the endogenous control.

### 2.6. Western Blotting

A lysis buffer was prepared (RIPA buffer: phenylmethanesulfonylfluoride (PMSF), 1 mM; Na3VO4, 2 mM; NaF, 100 mM) with a 1× protease inhibitor cocktail (Sigma, St Louis, MO, USA). Then, proteins were extracted from the neuroretinas in 80 µL of the aforementioned buffer. A total of 25 µg protein was resolved by 10% SDS-PAGE and transferred to a polyvinylidene difluoride (PVDF) membrane (Bio-Rad Laboratories, Madrid, Spain). The primary antibodies (Table 2) were incubated overnight at 4 °C.

The following day, the suitable horseradish peroxidase (HRP) secondary antibody anti-rabbit or anti-mouse (Dako Agilent, Santa Clara, CA, USA) was incubated for 1 h at room temperature. Anti-cyclophilin A (1:10,000; BML-SA296; Enzo, NY, USA) was used to normalize protein levels. Densitometric analysis of the western blot bands was performed with Image J software.

### 2.7. Immunohistochemical Analysis

Firstly, paraffined sections were deparaffinized in xylene and rehydrated in ethanol. Sections were fixed in acid methanol (−20 °C) for 1 minute and washed with 0.01M 4 phosphate buffered saline (PBS) at pH 7.4. Then, sections were incubated in blocking solution (3% bovine serum albumin (BSA), Tween 0.05% PBS) for 1 h at room temperature and afterwards, they were incubated overnight at 4 °C with specific primary antibodies (Table 3).

The following day, after washing, sections were incubated with a fluorescent ALEXA 488 or 594 as a secondary antibody (anti-rabbit or anti-mouse) (Life Technologies S.A, Madrid, Spain) in blocking solution for 1 h and washed again. Nuclei were counterstained using Hoechst 33342 (bisbenzimide), an organic compound that emits blue fluorescence when bound to DNA (Thermo Fisher Scientific, Eugene, OR, USA). Finally, samples were mounted in Mounting Medium Fluorescence (Prolong, Invitrogen, Thermo Fisher Scientific, Eugene, OR, USA) with a coverslip. Images were acquired with a confocal laser scanning microscope (FV1000; Olympus, Hamburg, Germany) at a resolution of 1024 × 1024 pixels. Five fields (three corresponding to the central and two to the peripheral retina) from each section were analyzed using ImageJ software (National Institutes of Health, Bethesda, MD, USA).

In order to evaluate glial reactivity, the degree of glial activation was analyzed using a scoring system based on the extent of GFAP (glial fibrillary acidic protein) staining previously described [11]. This scoring system (Table 4) has been used previously by our group [8,9,12]. The fluorescence intensity of the images was quantified by ImageJ.

For apoptosis assessment, the TUNEL (Terminal Transferase dUTP Nick-End Labeling) DeadEnd Fluorometric System kit (PROMEGA, Madison, WI, USA) was carried out using 488 nm as excitation wavelength and the range for detecting positive cells in the confocal laser scanning microscope was 515–565 nm (green). Finally, the number of green positive cells and total cells (in blue) was counted.

In order to evaluate cell proliferation, Ki67 positive cells were counted. In addition, co-labeling immunofluorescence combining Ki67 and NeuN (as a neuron specific marker) and CD-31 (as a marker for endothelium of the blood vessels) was performed.

To assess the nuclear translocation of NFκB, Z-series stacks of retinal section were captured at 1024 × 1024 pixels and orthogonal projections of z-stack were evaluated using laser confocal microscopy (FluoView ASW 1.4, Olympus, Hamburg, Germany).

### 2.8. Retinal Vascular Permeability

The permeability of retinal vasculature was examined ex vivo by assessing albumin leakage from the blood vessels into the retina using the Evans Blue albumin method. For this purpose, three animals per group were intravenously injected in the tail with a solution of Evans Blue (E2129 SIGMA, Sant Louis, MO, USA) (5 mg/mL dissolved in PBS pH 7.4). After injection, the animals turned blue, confirming dye uptake and distribution. After 2h, the mice were euthanized by cervical dislocation and the eyes were enucleated. The retinas of each animal (*n* = 3 per group) were isolated, weighed and rapidly protected from light. Flat-mounted slides were obtained, and cover slipped with a drop of the mounting medium Prolong Gold antifade reagent (Invitrogen, Thermo Fisher Scientific, Eugene, OR, USA). Digital images from different random fields of all retinas were acquired using a confocal laser scanning microscope (FV1000; Olympus, Hamburg, Germany) at ×60 using the 561-nm laser line, and each image was recorded with identical beam intensity at a size of 1024 pixels × 1024 pixels. For quantitative analysis of the albumin-bound Evans Blue, the number of extravasations per field of ×60 was counted. We used these images to assess the number of capillary branches and sprouts following a previously reported method [13].

### 2.9. Statistical Analysis

Comparisons of continuous variables were performed using ANOVA and the Bonferroni Post Hoc test with SPSS software (SPSS/Windows version 18; SPSS, Chicago, IL, USA). The statistical significance level was set at *p* < 0.05 (*).

## 3. Results

### 3.1. Topical Administration of Glucagon-Like Peptide-1 (GLP-1) Ameliorates Electroretinogram Abnormalities Induced by Diabetes

**Figure 1:**
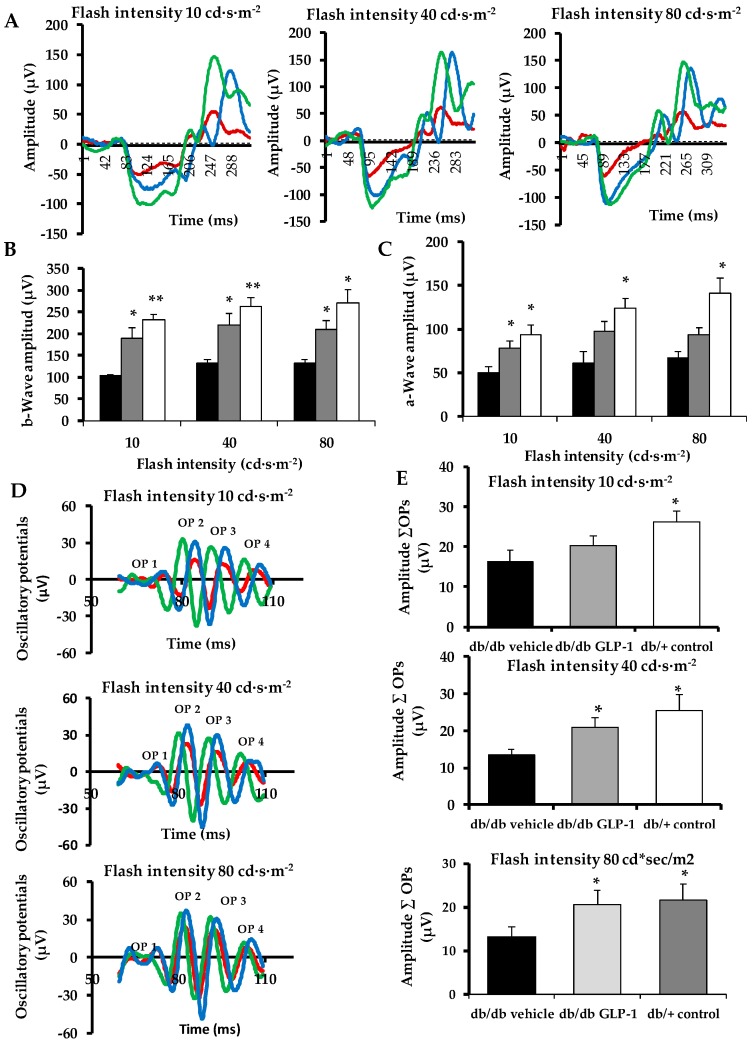
Electroretinogram. (**A**) Electroretinogram traces in response to low, medium and high stimulus intensities (10 cd·s·m^−2^, 40 cd·s·m^−2^ and 80 cd·s·m^−2^) in a representative non-diabetic mouse (green), a db/db mouse treated with vehicle (red) and a db/db mouse treated with glucagon-like peptide-1 (GLP-1) eye-drops (blue); (**B**) Quantitative analyses of b-wave amplitude in db/db mice treated with vehicle (black bars), db/db mice treated with GLP-1 eye drops (grey bars) and non-diabetic mice (white bars). (*n* = 4 mice per group) * *p* < 0.05 in comparison with db/db vehicle; ** *p* < 0.01 in comparison with db/db vehicle. (**C**) Quantitative analyses of a-wave amplitude in db/db mice treated with vehicle (black bars), db/db mice treated with GLP-1 eye drops (grey bars) and non-diabetic mice (white bars). (*n* = 4 mice per group) * *p* < 0.05 in comparison with db/db vehicle; (**D**) Oscillatory potential traces in response to low, medium and high stimulus intensities (10 cd·s·m^−2^, 40 cd·s·m^−2^ and 80 cd·s·m^−2^) in a representative non-diabetic mouse (green), a db/db mouse treated with vehicle (red) and a db/db mouse treated with GLP-1 eye-drops (blue); (**E**) Quantitative analyses of oscillatory potentials at 10 cd·s·m^−2^, 40 cd·s·m^−2^ and 80 cd·s·m^−2^ in db/db mice treated with vehicle (black bars), db/db mice treated with GLP-1 eye drops (grey bars) and non-diabetic mice (white bars). (*n* = 4 mice per group) * *p* < 0.05 in comparison with db/db vehicle.

The amplitude of the b-wave, which is predominantly produced by Müller and bipolar cells, was significantly lower in diabetic mice in comparison with non-diabetic mice (Figure 1A,B). Treatment with GLP-1 was able to ameliorate these functional abnormalities induced by diabetes. The a-wave, which is derived from photoreceptor function, was also significantly lower in diabetic mice treated with vehicle than in non-diabetic mice at all intensities (Figure 1A,C). The amplitude of a-wave in the diabetic mice treated with GLP-1 was significantly higher than in diabetic mice treated with vehicle at 10 cd·s·m^−2^ flash intensity. In addition, GLP-1 treatment ameliorated the amplitude of oscillatory potentials (OPs), which reflect the neuronal synaptic activity of amacrine cells and other neurons of the inner retina (Figure 1D,E).

### 3.2. Neurodegeneration Is Inhibited by Glucagon-Like Peptide-1 (GLP-1) Eye-Drops in Diabetic Mice

**Figure 2:**
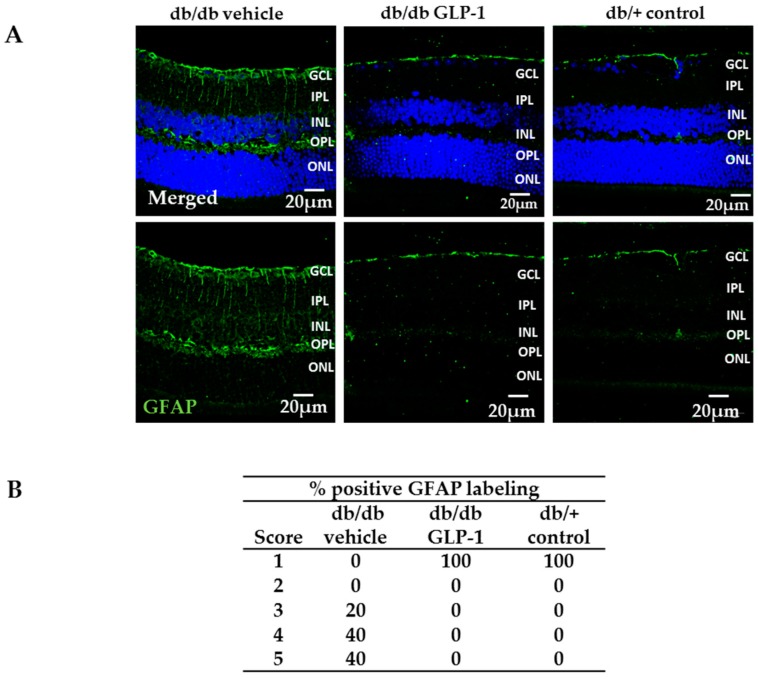
Glial activation. (**A**) Comparison of glial fibrillary acidic protein (GFAP) immunoreactivity (green) among representative samples from diabetic mice treated with vehicle or glucagon-like peptide-1 (GLP-1) eye-drops and from a non-diabetic mouse. Scale bars, 20 µm; (**B**) Quantification of glial activation based on the extent of GFAP staining. *n* = 4 mice per group (five retinal sections per mouse).

In non-diabetic mice, GFAP expression was confined to the retinal ganglion cell layer (GCL), and therefore, the GFAP score was <2 (Figure 2A,B). By contrast, diabetic mice treated with vehicle presented an aberrant GFAP extent, this being the GFAP score ≥3, which was significantly higher than in non-diabetic mice (Figure 2A,B). Eye drops containing GLP-1 led to a significant decrease of glial activation (GFAP score ≤2) (Figure 2A,B).

**Figure 3:**
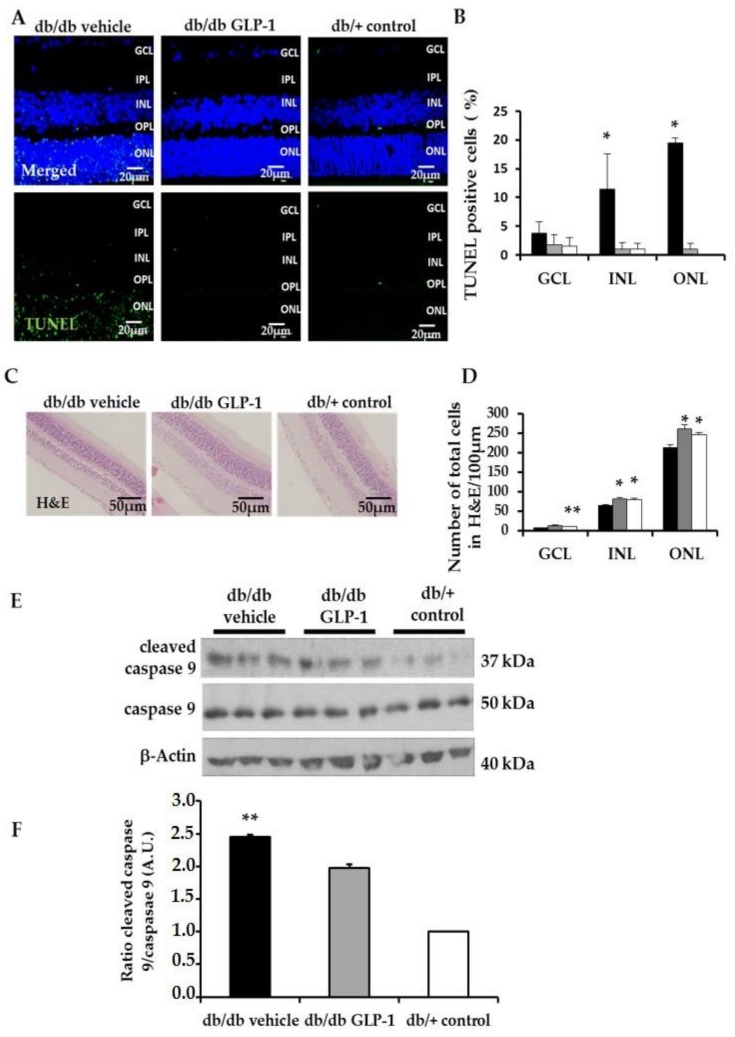
(**A**) Terminal Transferase dUTP Nick-End Labeling (TUNEL) positive immunofluorescence (green) in a representative db/db mouse treated with vehicle, a db/ db mouse treated with glucagon-like peptide-1 (GLP-1) eye-drops and a non-diabetic mouse. Nuclei were labelled with Hoechst stain (blue). Scale bars, 20 µm; (**B**) Quantification of TUNEL-positive cells in the neuroretina. Results are means expressed as a percentage. *n* = 4 mice per group. * *p* < 0.001 in comparison with the other groups; (**C**) Comparison of hematoxylin-eosin retinas among representative samples from diabetic mice treated with vehicle or GLP-1 eye-drops and from a non-diabetic mouse. Scale bars, 50 µm. *n* = 3; (**D**) Quantification of the number of total cells in ganglion cell layer (GCL), inner nuclear layer (INL) and outer nuclear layer (ONL) calculated in hematoxylin-eosin staining. *n* = 3 mice per groups; * *p* < 0.05 db/db vehicle in comparison with other groups, ** *p* < 0.01 db/db vehicle in comparison with other groups; (**E**) Western blotting bands of cleaved and total caspase 9 and (**F**) densitometric analyses of retinas from db/db mice treated with vehicle (black bars), db/db mice treated with GLP-1 eye drops (grey bars) and non-diabetic mice (white bars) *n* = 3 * *p* < 0.05 db/db vehicle in comparison with the other groups; GCL, ganglion cell layer; INL, inner nuclear layer; IPL, inner plexiform layer; ONL, outer nuclear layer; OPL, outer plexiform layer.

Retinal apoptosis, analyzed by TUNEL, was significantly higher in diabetic mice treated with vehicle than in non-diabetic mice in the inner (INL) and outer nuclear layer (ONL) (Figure 3A,B). The treatment with GLP-1 led to a significant decrease of apoptosis in the aforementioned nuclear layers (Figure 3A,B) and also in the ratio of cleaved caspase 9/total caspase 9, thus supporting the anti-apoptotic effects observed in TUNEL assay (Figure 3E,F).

### 3.3. Topical Administration of Glucagon-Like Peptide-1 (GLP-1) Preserves Retinal Thickness and Promotes Neurogenesis

**Figure 4:**
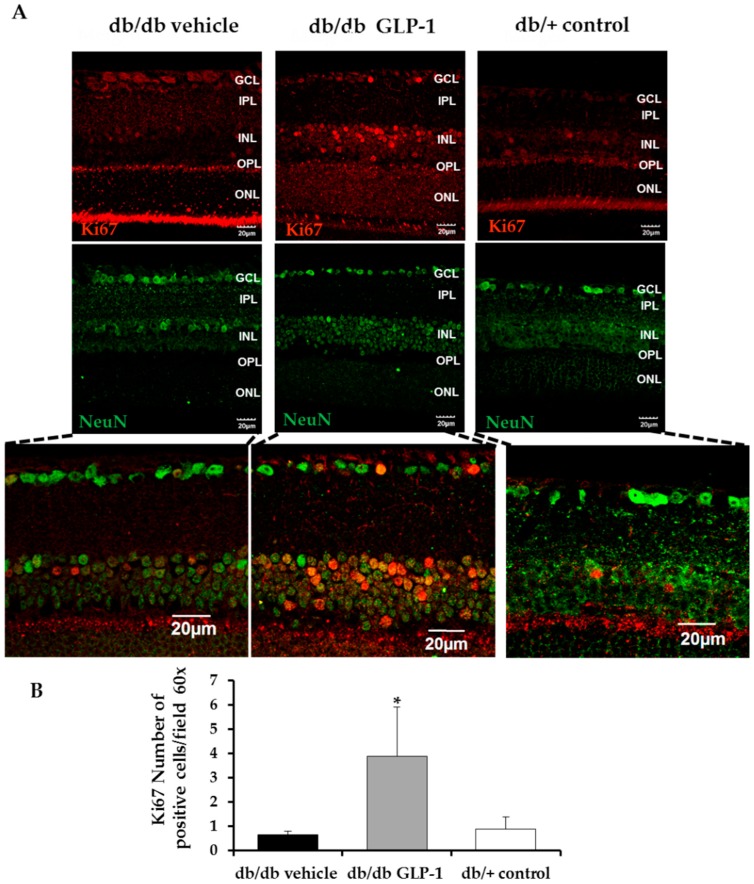
(**A**) Comparison of Ki67 (red) immunofluorescence colabeling with NeuN (neuron specific marker) (green) in retinas among representative samples from diabetic mice treated with vehicle or glucagon-like peptide-1 (GLP-1) eye-drops and from a non-diabetic mouse. Scale bars, 20 µm. *n* = 4; (**B**) Quantification of Ki67-positive cells per ×60 field. *n* = 4 mice per groups; * *p* < 0.05 db/db GLP-1 in comparison with other groups; GCL, ganglion cell layer; INL, inner nuclear layer; IPL, inner plexiform layer; ONL, outer nuclear layer; OPL, outer plexiform layer.

As expected, the numbers of total cells in GCL, INL, and ONL were lower in diabetic mice than in non-diabetic mice. The number of retinal cells detected in diabetic mice treated with GLP-1 was similar to that the observed in non-diabetic mice (Figure 3C,D). These results suggest that topical administration of GLP-1 could promote neurogenesis. In this regard, Ki67, a marker of proliferation, was significantly increased in GLP-1 treated diabetic mice in comparison with the other groups (Figure 4A,B).

### 3.4. Treatment with Glucagon-Like Peptide-1 (GLP-1) down-Regulates Inflammation Induced by Diabetes through the Inhibition of NF-κB, Proinflammatory Cytokines and the NLRP3 Inflammasome Pathway

**Figure 5:**
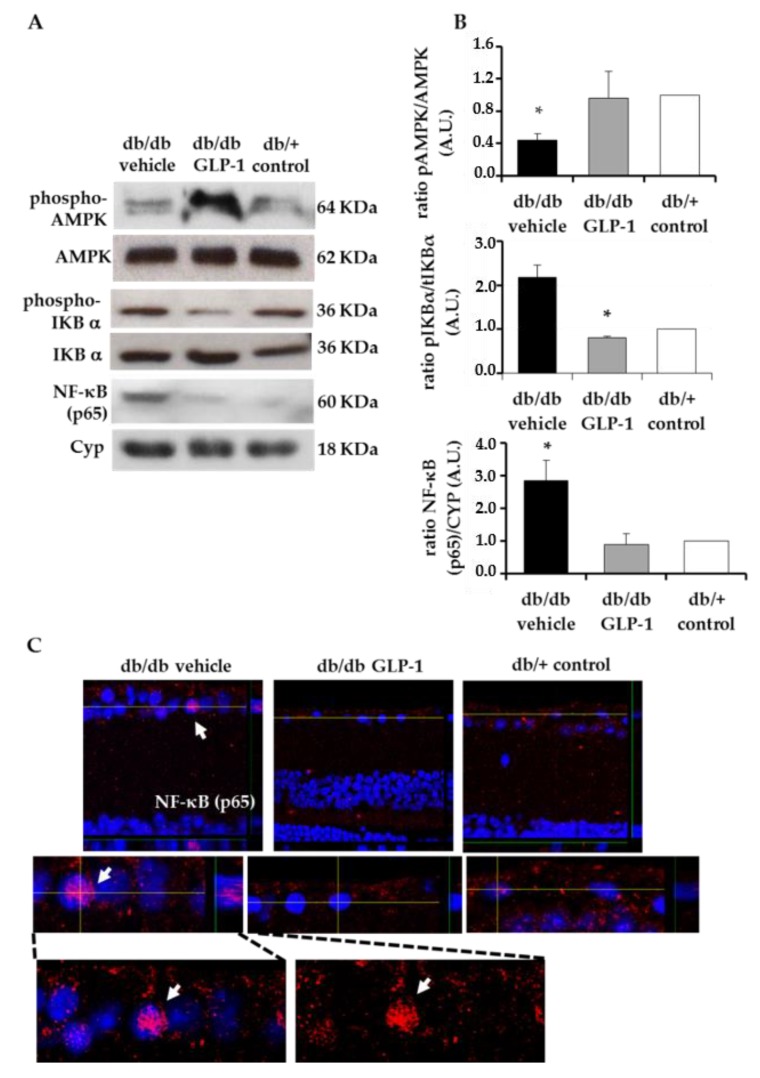
Anti-inflammatory effects. (**A**) Western blotting bands of phospho-AMPK, total AMPK, phospho-IKBα, total IKBα, NF-κB (p65) and (**B**) densitometric analyses in mouse retinas in db/db mice treated with vehicle (black bars), db/db mice treated with glucagon-like peptide-1 (GLP-1) eye drops (grey bars) and non-diabetic mice (white bars) *n* = 4 * *p* < 0.05 db/db vehicle in comparison with other groups, (**C**) Orthogonal view of confocal images from NF-kB p65 in a representative db/db mouse treated with vehicle, a db/db mouse treated with GLP-1 eye-drops and a non-diabetic mouse by immunofluorescence. Red: NF-kB p65 antibody stained. Blue: Hoechst stained.

We found that topical administration of GLP-1 down-regulated the phosphorylation of IKBα induced by diabetes, thus producing a down-regulation of NF-κB in the retina. The ratio phospho-IKBα/total-IKBα was significantly lower in retinas from diabetic mice treated with GLP-1 than in retinas from diabetic mice treated with vehicle, and similar to non-diabetic mice (Figure 5A,B). Consequently, diabetic mice treated with GLP-1 exhibited a significantly lower content of active NF-κB (p65) protein than diabetic mice treated with vehicle and similar to non-diabetic mice (Figure 5A,B). Immunofluorescence analysis also confirmed that the active NF-κB (p65) translocated to the nucleus in diabetic retina. Treatment with GLP-1 also reduced nuclear translocation of NF-κB (p65) in retinal cells (Figure 5C).

In addition, we observed that activation of the AMPK signaling pathway was one of the mechanisms involved in NF-κB inhibition by GLP-1. Thus, retinas from db/db mice treated with GLP-1 eye-drops exhibited a higher ratio phospho-AMPK/ total-AMPK than retinas from db/db mice treated with vehicle (Figure 5A,B).

**Figure 6:**
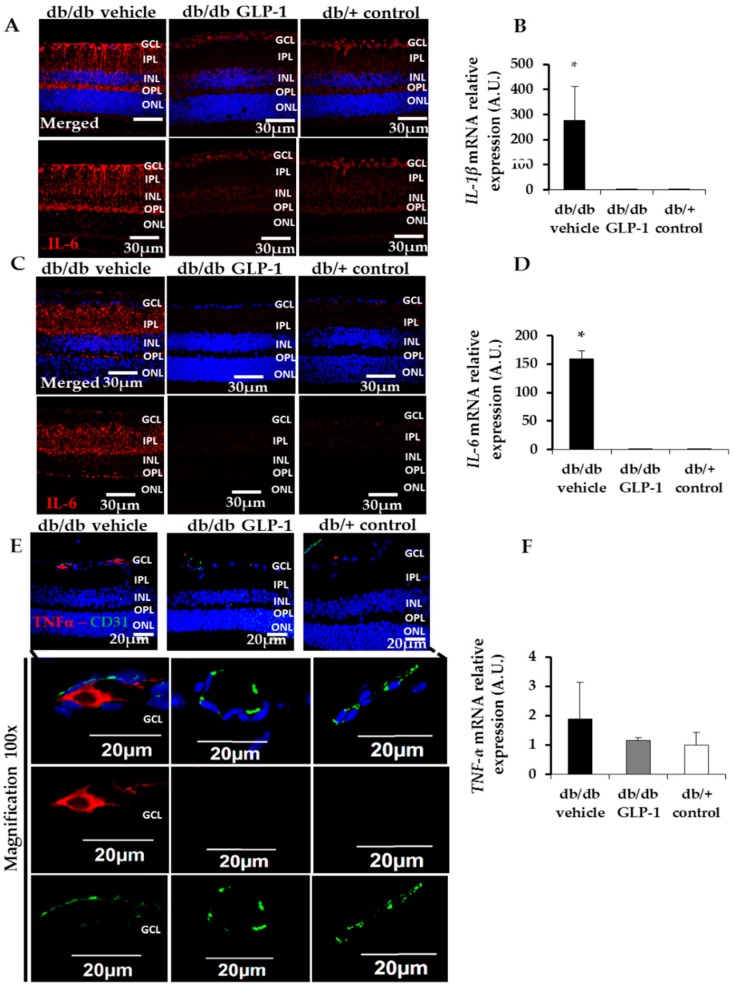
(**A**) Immunofluorescence of IL-1β. Scale bars, 30 µm. Nuclei were labelled with Hoechst stain (blue); (**B**) Real-time quantitative reverse transcription polymerase chain reaction (RT-PCR) analysis of IL-1β in db/db mice treated with vehicle (black bars), db/db mice treated with glucagon-like peptide-1 (GLP-1) eye drops (grey bars) and non-diabetic mice (white bars), *n* = 4. * *p* < 0.01 db/db vehicle in comparison with other groups; (**C**) Immunofluorescence of IL-6. Scale bars, 30 µm. Nuclei were labelled with Hoechst stain (blue); (**D**) Real-time quantitative RT-PCR analysis of IL-6 in db/db mice treated with vehicle (black bars), db/db mice treated with GLP-1 eye drops (grey bars) and non-diabetic mice (white bars), *n* = 4. * *p* < 0.01 db/db vehicle in comparison with other groups; (**E**) Immunofluorescence of colabelling TNF-α/CD31. Scale bars, 30 µm. Nuclei were labelled with Hoechst stain (blue); (**F**) Real-time quantitative RT-PCR analysis of TNF-α in db/db mice treated with vehicle (black bars), db/db mice treated with GLP-1 eye drops (grey bars) and non-diabetic mice (white bars) *n* = 4. GCL, ganglion cell layer; INL, inner nuclear layer; IPL, inner plexiform layer; ONL, outer nuclear layer; OPL, outer plexiform layer.

As a result of NF-κB activation induced by diabetes, retinas from diabetic mice treated with vehicle showed a significant upregulation of several proinflammatory cytokines (mRNA and protein) such as IL-1β and IL-6 (Figure 6A–D). Topical treatment with GLP-1 dramatically abrogated this upregulation, leading to levels like the non-diabetic control group (Figure 6A–D). TNFα was also increased in retinas from diabetic mice in comparison with control mice, and a lower abundancy was found in the group of mice treated with GLP-1 (Figure 6E,F). However, in both cases the differences did not reach statistical significance.

**Figure 7:**
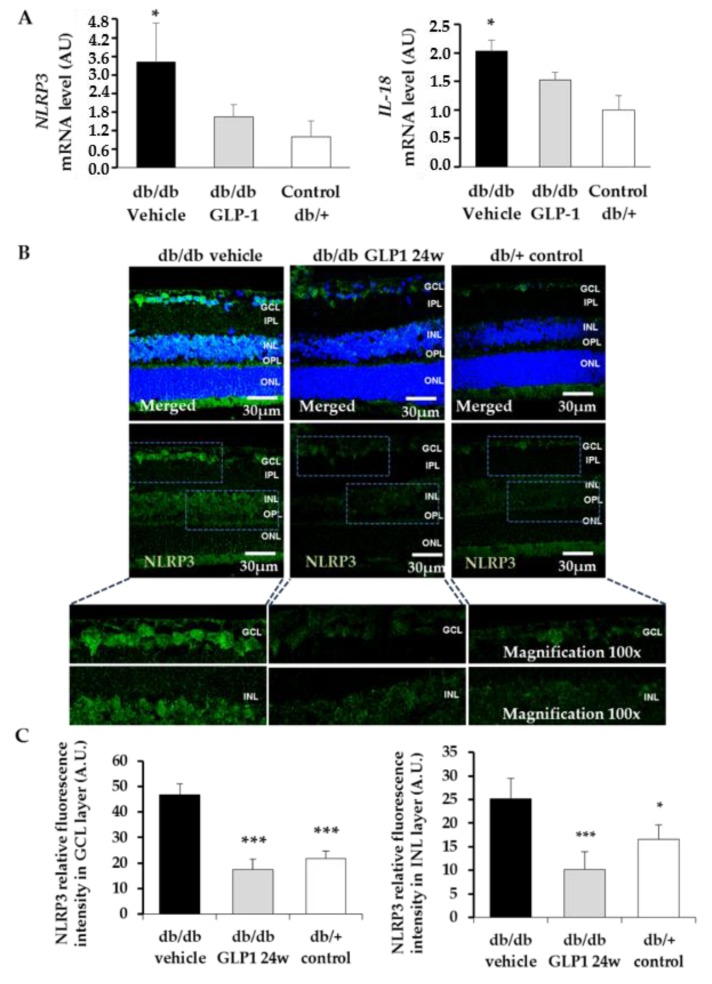
(**A**) Real-time quantitative reverse transcription polymerase chain reaction (RT-PCR) analysis of NLRP3 and IL-18 in db/db mice treated with vehicle (black bars), db/db mice treated with glucagon-like peptide-1 (GLP-1) eye drops (grey bars) and non-diabetic mice (white bars) *n* = 4 * *p* < 0.05 db/db vehicle in comparison with other groups; (**B**) Comparison of NLRP3 immunoreactivity (red) among representative samples from diabetic mice treated with vehicle or GLP-1 eye-drops and from a non-diabetic mouse and quantification of the immunofluorescence (**C**). Nuclei were labelled with Hoechst stain (blue). Scale bars, 30 µm; *n* = 4 * *p* < 0.05 in comparison with db/db GLP-1; *** *p* < 0.001 in comparison with db/db vehicle. GCL, ganglion cell layer; INL, inner nuclear layer; IPL, inner plexiform layer; ONL, outer nuclear layer; OPL, outer plexiform layer.

Finally, we observed a significant upregulation of NLRP3 inflammasome pathway in db/db mice treated with vehicle. GLP-1 treatment was able to significantly inhibit the activation of NLRP3 (mRNA and protein) (Figure 7A–C), as well as the overexpression of IL-18, a cytokine triggered by NLRP3 (Figure 7A).

### 3.5. Treatment with Glucagon-Like Peptide-1 (GLP-1) up-Regulates Survival Pathways in the Diabetic Retina

**Figure 8:**
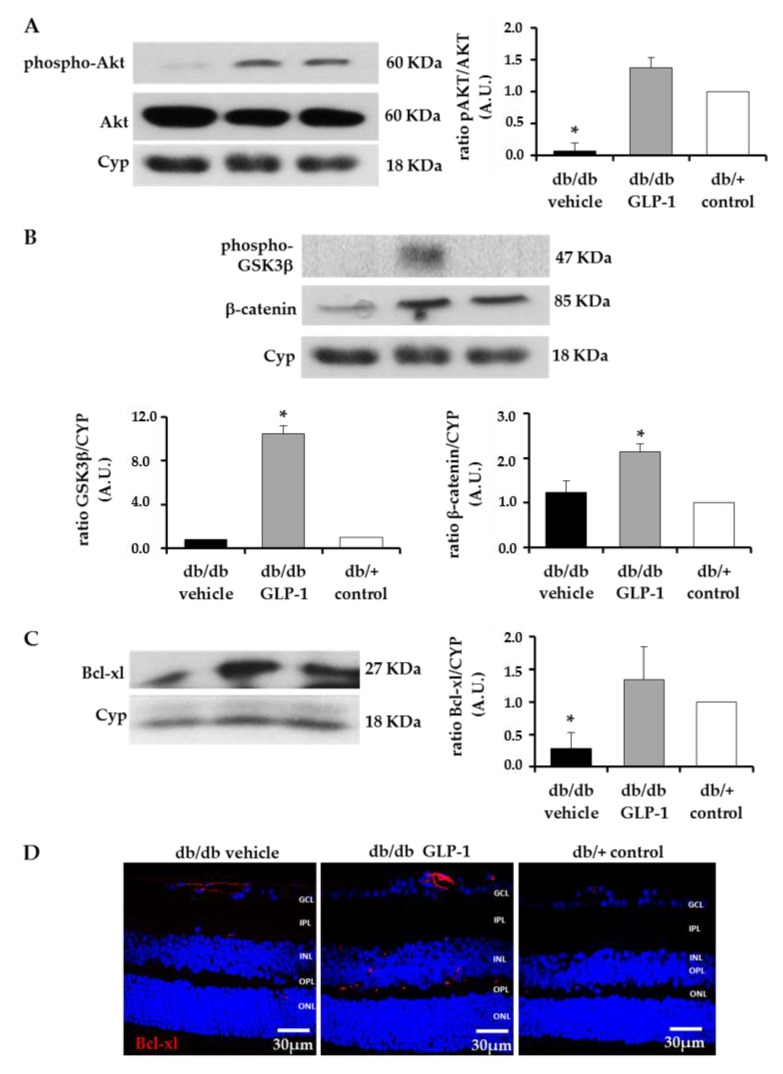
Survival pathway. (**A**) Western blotting bands of phospho-Akt/Akt and densitometric analyses in mouse retinas in db/db mice treated with vehicle (black bars), db/db mice treated with glucagon-like peptide-1 (GLP-1) eye drops (grey bars) and non-diabetic mice (white bars) *n* = 4 * *p* < 0.05 in comparison with the other groups; (**B**) Western blotting bands of phospho-GSK3β and β-catenin. Densitometric analyses in mouse retinas in db/db mice treated with vehicle (black bars), db/db mice treated with GLP-1 eye drops (grey bars) and non-diabetic mice (white bars) *n* = 4 * *p* < 0.05 in comparison with the other groups; (**C**) Western blotting bands of B-cell lymphoma-extra-large protein (Bcl-xL) and densitometric analyses in mouse retinas in db/db mice treated with vehicle (black bars), db/db mice treated with GLP-1 eye drops (grey bars) and non-diabetic mice (white bars) *n* = 4 * *p* < 0.05 in comparison with the other groups; (**D**) Comparison of Bcl-xL immunoreactivity (red) among representative samples from diabetic mice treated with vehicle or GLP-1 eye-drops and from a non-diabetic mouse. Scale bars, 30 µm; *n* = 4. Cyp, ciclophyline.

Topical GLP-1 treatment was able to inhibit the downregulation of the ratio phospho-Akt/total Akt also in db/db mice (Figure 8A). Notably, the retinas of diabetic mice treated with GLP-1 showed a significantly increased expression of phospho-GSK3 β and β-catenin in comparison with diabetic mice treated with vehicle and non-diabetic mice (Figure 8B). In addition, topical administration of GLP-1 was able to significantly reduce the downregulation of anti-apoptotic transmembrane molecule B-cell lymphoma-extra-large protein (Bcl-xL) induced by diabetes (Figure 8C,D).

### 3.6. Treatment with Glucagon-Like Peptide-1 (GLP-1) Reduces Diabetes-Induced VEGF Overexpression and Vascular Leakage

**Figure 9:**
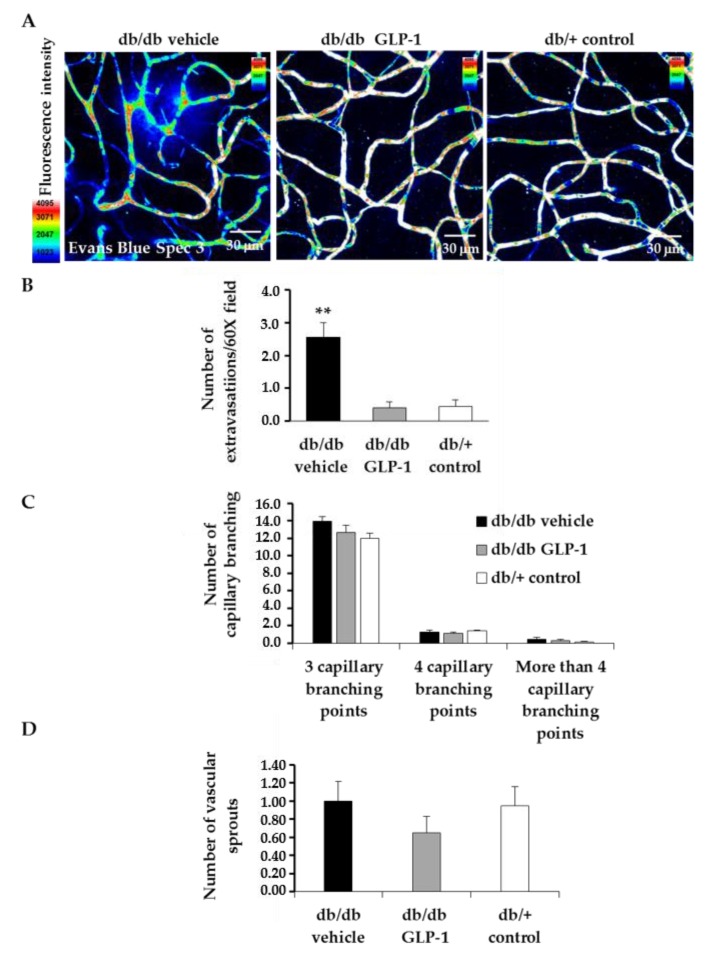
Microvascular abnormalities. (**A**) Confocal immunofluorescence images of vascular permeability assessed by Evans Blue dye leakage in retinal whole mounts. Spec3, fluorescent spectral signature 3. Scale bars, 30 µm; (**B**) For quantification, the number of extravasations per field of 60× retina was counted. * *p* < 0.01 in comparison with the other groups; (**C**) The number of capillary branching and (**D**) The number of vascular sprouts in db/db mice treated with vehicle (black bars), db/db mice treated with glucagon-like peptide-1 (GLP-1) eye drops (grey bars) and non-diabetic mice (white bars). *n* = 4 per group, 25–30 fields per animal were analyzed.

**Figure 10:**
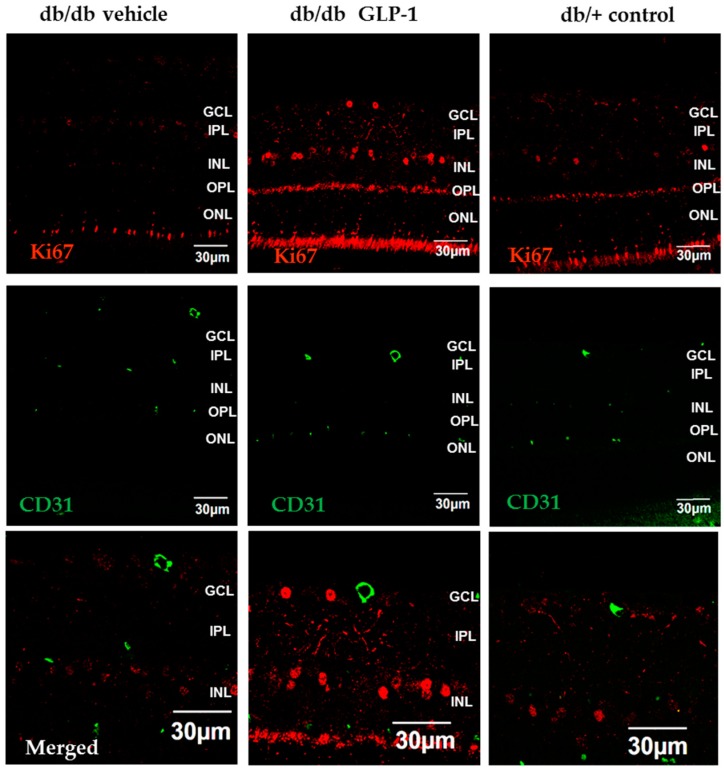
Ki67 (red) immunofluorescence co-labeling with CD-31 (green) in retinas of representative samples from diabetic mice treated with vehicle or glucagon-like peptide-1 (GLP-1) eye-drops, and from a non-diabetic mouse. Scale bars, 30 µm. *n* = 4; GCL, ganglion cell layer; INL, inner nuclear layer; IPL, inner plexiform layer; ONL, outer nuclear layer; OPL, outer plexiform layer.

We identified extravascular location of Evans Blue in the diabetic mice retina (Figure 8A,B). Albumin leakage was significantly lower in diabetic mice treated with topical administration of GLP-1 than in diabetic mice treated with the other groups (Figure 9A,B). The number of capillary branching points and sprouts, as well as the intercapillary distances, were comparable among all the groups (Figure 9C,D). GLP-1 administration did not increase CD-31 inmmunostaining. In addition, we did not observe any co-localization between CD-31 and Ki67 (Figure 10).

**Figure 11:**
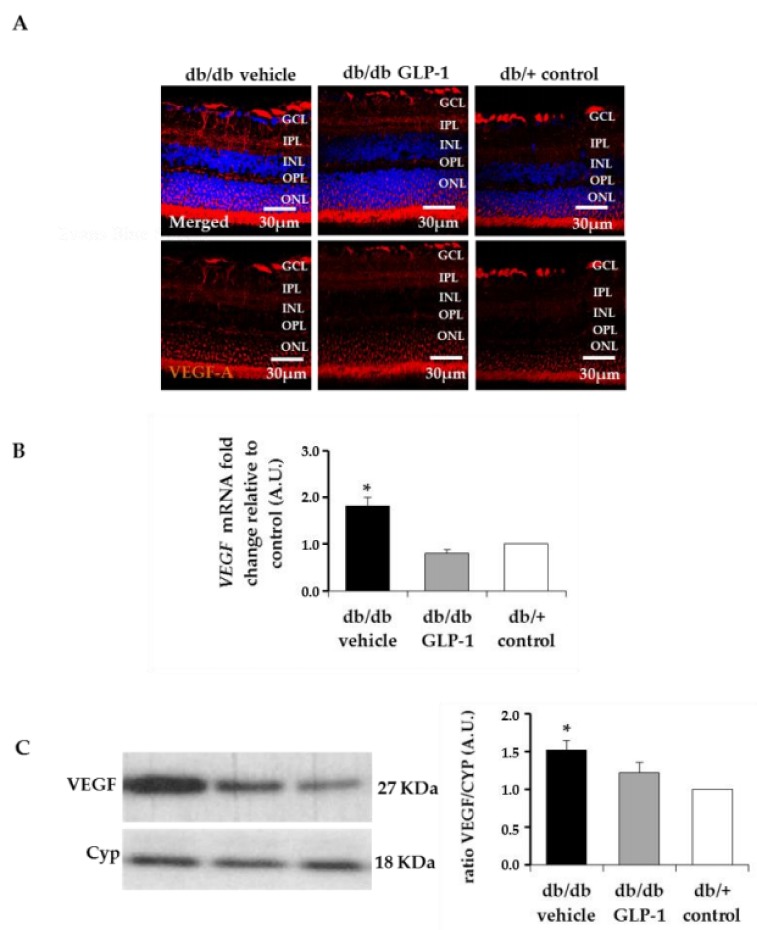
(**A**) Immunofluorescence of VEGF. Scale bars, 30 µm. Nuclei were labelled with Hoechst stain (blue); (**B**) Real-time quantitative reverse transcription polymerase chain reaction (RT-PCR) analysis of VEGF A in db/db mice treated with vehicle (black bars), db/db mice treated with glucagon-like peptide-1 (GLP-1) eye drops (grey bars) and non-diabetic mice (white bars) *n* = 4 * *p* < 0.05 in comparison with the other groups; (**C**) Western blotting bands of VEGF and densitometric analyses in mouse retinas in db/db mice treated with vehicle (black bars), db/db mice treated with GLP-1 eye drops (grey bars) and non-diabetic mice (white bars) *n* = 4 * *p* < 0.05 in comparison with other groups; GCL, ganglion cell layer; INL, inner nuclear layer; IPL, inner plexiform layer; ONL, outer nuclear layer; OPL, outer plexiform layer.

Notably, mRNA levels of VEGF were significantly lower in diabetic mice treated with GLP-1 in comparison with diabetic mice treated with vehicle (Figure 11C). The protein levels of VEGF assessed by IF or Western blot were higher in diabetic mice than in non-diabetic mice. (Figure 11D,E). Moreover, diabetic mice treated with GLP-1 showed lower levels of VEGF than diabetic mice treated with vehicle assessed by Western blot (Figure 11E).

## 4. Discussion

In the present study we provide evidence that the topical administration of GLP-1 not only arrests the progression of neurodegeneration and vascular leakage, but even reverts these essential pathogenic events of DR. The main mechanisms involved are the downregulation of VEGF and, in particular, the anti-inflammatory action.

In a previous study we have already found that topical administration of GLP-1 inhibited the upregulation of VEGF induced by diabetes in db/db mice 12 weeks old after a short-term diabetes duration (around 8 weeks). In the present study, we have found that this effect persists at 24 weeks after 20 weeks of retinal exposure to the diabetic milieu, when structural and functional changes of NVU are well-established.

In addition, we have found that after 3 weeks of treatment, GLP-1 exerts a potent anti-inflammatory action through an inhibition of the NF-κB, IL-1β, IL-6, TNF-α, and NLRP3 inflammasome pathways. This later action resulted in a significant downregulation of IL-18. To the best of our knowledge, this pleiotropic action of GLP-1 based on a multifaceted and powerful anti-inflammatory effect has not previously been reported, and opens up a new avenue for topical GLP-1 administration in other eye diseases in which inflammation plays an important role.

It has been reported that liraglutide, a GLP-1R agonist, might exert an anti-inflammatory effect by inhibiting NF-κB through the activation of AMPK in human umbilical vein endothelial cells and in murine transformed endothelial cells (SVEC4) [14]. Moreover, the inhibitor of DPP-IV sitagliptin also inhibited NF-κB activating AMPK in a model of atherosclerosis in apolipoprotein-E-knockout mice [15] and in rats with streptozotocin-induced diabetes [16]. In agreement with these reports, our results indicate that eye drops containing GLP-1 were able to activate AMPK and inhibit NF-κB through a down-regulation of IKBα phosphorylation in the retina of a mouse model of spontaneous type 2 diabetes. This anti-inflammatory action can be envisaged as crucial for reverting structural changes such as glial activation and vascular leakage, as well as neurodysfunction assessed by ERG.

Apart from the downregulation of VEGF and pro-inflammatory mediators, GLP-1 exerts a significant effect in up-regulating survival pathways in the diabetic retina. We previously demonstrated that topical administration of liraglutide, a GLP-1RA, prevented the downregulation of BcL-xL and Akt induced by diabetes in a short-term period [8]. In the present study we have found that after 20 weeks of diabetes, topical administration of GLP-1 resulted in a significant increase not only of these two important neuronal survival factors but of the entire PI3K/Akt/GSK3B/β-catenin pathway. There is growing evidence suggesting that the activation of common pathways to insulin signaling, such as the Akt pathway, is essential for the survival of retinal neurons [17,18,19,20,21,22]. It was previously reported that Akt cascade activation confers neuroprotection by inhibiting the apoptosis of photoreceptors [23]. In fact, our ERG findings, showing an increase in the a-wave, support this mechanism of action. It should be noted that the upregulation of the Akt pathway led to an increase of IKBα, a specific endogenous inhibitor of NF-κB [24], thus linking prosurvival pathway activation with the anti-inflammatory activity induced by GLP-1. In addition, we observed that in the response of GLP-1 eye-drops, GSK3 is phosphorylated (Ser9) and β-catenin is increased. The stabilization of β-catenin leads to the translocation of the protein to the nucleus, where it activates genes related to cell survival and proliferation [25,26,27]. Interestingly, β-catenin plays a role in the process of adult neurogenesis promoting stem cell proliferation [28]. It has been suggested that increased adult neurogenesis by GLP-1RAs participates in functional recovery in animal models of brain disorders. Porter et al. [29] demonstrated that subcutaneous administration of liraglutide elicits synaptic plasticity and neurogenesis in the hippocampus of mice with severe obesity and insulin resistance. Darsalia et al. [30] reported that exendin-4 treatment increased neural stem cell proliferation in the striatum of type 2 diabetic Goto-Kakizaki rats after inducing stroke by transient middle cerebral artery occlusion. We have found that topical treatment with GLP-1 was able to prevent the thinning of the neuroretina induced by diabetes and restored the number of cells to the same level as that observed in non-diabetic mice. In addition, this effect was associated with a significant increase of Ki67, an excellent marker of cellular proliferation [31]. It could be argued that the cell proliferation could be of vascular rather than neural origin, but GLP-1 topically administered was unable to increase the number of capillary branching points and sprouts in db/db mice. In addition, GLP-1 administration did not increase CD-31 immunostaining and a co-localization between Ki67 and CD-31 was not observed. Taken together, all these findings suggest that GLP-1 is not only a neuroprotective agent but also that it could play an active role in neurogenesis and, therefore, might be useful in other retinal neurodegenerative diseases. However, further research to confirm our results in other experimental models and to elucidate the involved molecular mechanisms are needed.

Since GLP-1 crosses the blood-retinal barrier, one might wonder why we have performed the experiment using the topical route. There are several reasons for using this type of local administration. First, eye drops of GLP-1 are unable to significantly pass over the systemic circulation and so decrease blood glucose levels. Hence, all the effects observed in the GLP-1 treated group are direct effects of GLP-1 at retinal level and unrelated to its capacity to increase systemic insulin secretion or to lower blood glucose levels. In fact, the lowering of blood glucose levels is an important limiting factor in the design of any clinical trial specifically addressed to test the eventual beneficial effects of GLP-1RAs or other antidiabetic agents on the diabetic retina. This is because in the case of beneficial results in preventing DR or arresting its progression being obtained, it would be very difficult to know whether they could be due to the improvement of diabetes control or to the drug itself. Other important reasons in the clinical setting for the use of topical GLP-1 administration is the possibility of its being self-administered and its limited action in the eye, thus minimizing the associated systemic effects. Therefore, it seems reasonable to hypothesize that eye drops of GLP-1RAs could be used for DR treatment in most patients with diabetes, including those patients in whom the systemic administration of GLP-1RAs is not recommended (i.e., patients within the metabolic objectives under treatment with metformin, those experiencing GLP-1RAs related adverse gastrointestinal effects, patients with type 1 diabetes or patients with type 2 diabetes with clear insulinopenia). However, specific clinical trials aimed at exploring the safety and effectiveness of the topical administration of GLP-1 for treating DR are needed.

In conclusion, the topical administration of GLP-1 reverts the impairment of the neurovascular unit induced by diabetes. Our results suggest that neuroprotective and anti-inflammatory properties are essential in accounting for the effectiveness of GLP-1 for treating DR. The complexity of the mechanisms involved in the pathogenesis of DR suggests that those treatments targeting multiple pathways, such as GLP-1, could be more effective than those blocking a single pathogenic mechanism.

## Figures and Tables

**Table 1:** Primers used for RT-PCR.

Gen Symbol	Sequence Primer
IL-1 Forward (5′-3′)	5′-GCAACTGTTCCTGAACTCAACT-3′
IL-1 Reverse (5′-3′)	5′-ATCTTTTGGGGTCCGTCAACT-3′
IL-6 Forward (5′-3′)	5′-TAGTCCTTCCTACCCCAATTTCC-3′
IL-6 Reverse (5′-3′)	5′-TTGGTCCTTAGCCACTCCTTC-3′
TNF Forward (5′-3′)	5′-CCCTCACACTCAGATCATCTTCT-3′
TNF Reverse (5′-3′)	5′-GCTACGACGTGGGCTACAG-3′
VEGFa Forward (5′-3′)	5′-GAGTACCCCGACGAGATAGA-3′
VEGFa Reverse (5′-3′)	5′-GGCTTTGGTGAGGTTTGAT-3′
NLRP-3 Forward (5′-3′)	5′-ATTACCCGCCCGAGAAAGG-3′
NLRP-3 Reverse (5′-3′)	5′-TCGCAGCAAAGATCCACACAG-3′
IL-18 Forward (5′-3′)	5′-AGCAGTCCCAACTAAGCAGTA-3′
IL-18 Reverse (5′-3′)	5′-CAGCCAGTAGAGGATGCTGA-3′
Actin Forward (5′-3′)	5′-CTAAGGCCAACCGTGAAAG-3′
Actin Reverse (5′-3′)	5′-ACCAGAGGCATACAGGGACA-3′

**Table 2:** Primary antibodies and specific dilutions used in Western Blot analysis.

Antibody	Description
p-AMPK	1:1000; ab80039; Abcam, Cambridge, UK
AMPK	1:1000; ab133448; Abcam, Cambridge, UK
p-IKBα	1:1000; ab133462; Abcam, Cambridge, UK
IKBα	1:5000; ab32518; Abcam, Cambridge, UK
NF-κB (p65)	1:1000; sc-8008; Santa Cruz, Dallas, Texas, USA
p-AKT	1:1000; #2965; Cell Signaling, Leiden, The Netherlands
AKT	1:5000; #9272; Cell Signaling, Leiden, The Netherlands
p-GSK3β	1:1000; ab75745; Abcam, Cambridge, UK
β-catenin	1:1000; GTX132611; Genetex, California, USA
Bcl-xL	1:1000; 610211; BD Biosciences, Sparks, MD, USA
VEGF	1:5000; ab46154; Abcam, Cambridge, UK

**Table 3:** Targets, dilution, and sources of applied primary antibodies in immunofluorescence.

Antibody	Description
GFAP	rabbit monoclonal; 1:500; ab7260 (Abcam, Cambridge, UK)
Ki67	rabbit polyclonal; 1:500; ab15580 (Abcam, Cambridge, UK)
NFκB	mouse monoclonal; 1:100; sc-8008 (Santa Cruz, Dallas, Texas, USA)
IL-1β	rabbit polyclonal; 1:100; ab9722 (Abcam, Cambridge, UK)
IL-6	rabbit polyclonal; 1:100; ab7737 (Abcam, Cambridge, UK)
TNF-α	mouse monoclonal; 1:100; ab8348 (Abcam, Cambridge, UK)
Collagen IV	rabbit polyclonal; 1:200; ab6586 (Abcam, Cambridge, UK)
NLRP3	rabbit polyclonal; 1:200; ab214185 (Abcam, Cambridge, UK)
Bcl-xL	rabbit polyclonal; 1:100; 610211 (BD Biosciences, Sparks, MD, USA)
VEGFA	rabbit monoclonal; 1:100; ab52917 (Abcam, Cambridge, UK)

GFAP: Glial fibrillary acidic protein; NFkB: Nuclear factor kappa B; IL: Interleukin; TNF-α: Tumor necrosis factor α; NLRP3: NLR family pyrin domain containing 3; Bcl-xL: B-cell lymphoma-extra large; VEGFA: vascular endothelial growth factor A.

**Table 4:** Scoring system to evaluate glial activation.

Glial Fibrillary Acidic Protein (GFAP) Score	Description
1	Müller cell endfeet region/GCL only
2	Müller cell endfeet region/GCL plus a few proximal processes
3	Müller cell endfeet plus many processes, but not extending to ONL
4	Müller cell endfeet plus processes throughout with some in the ONL
5	Müller cell endfeet plus lots of dark processes from GCL to the outer margin of ONL

## Abbreviations

BRB	Blood retinal barrier
DR	Diabetic retinopathy
NPDR	Non-proliferative Diabetic retinopathy
GFAP	Glial fibrillar acidic protein
GLAST	Glutamate−aspartate transporter
GLP-1	Glucagon-like peptide 1
GLP1-R	Glucagon-like peptide 1 receptor
GLP-1RA	Glucagon-like peptide 1 receptor agonist
